# Polymorphisms in MicroRNA Genes And Genes Involving in NMDAR Signaling and Schizophrenia: A Case-Control Study in Chinese Han Population

**DOI:** 10.1038/srep12984

**Published:** 2015-08-10

**Authors:** Yanxia Zhang, Mei Fan, Qingzhong Wang, Guang He, Yingmei Fu, Huafang Li, Shunying Yu

**Affiliations:** 1 Shanghai Key Laboratory of Psychotic Disorders, Shanghai Mental Health Center, Shanghai Jiao Tong University School of Medicine, 600 Wan Ping Nan Road, Shanghai 200030, China; 2 Bio-X Institutes, Key Laboratory for the Genetics of Developmental and Neuropsychiatric Disorders (Ministry of Education), Shanghai Jiao Tong University, 600 Wan Ping Nan Road, Shanghai 200030, China; 3 Institution of Drug Clinical Trial, Shanghai Mental Health Center, Shanghai Jiao Tong University School of Medicine, 600 Wan Ping Nan Road, Shanghai 200030, China

## Abstract

Disturbances in glutamate signaling caused by disruption of N-methyl-D-aspartate-type glutamate receptor (NMDAR) have been implicated in schizophrenia. Findings suggested that miR-219, miR-132 and miR-107 could involve in NMDAR signaling by influencing the expression of pathway genes or the signaling transmission and single nucleotide polymorphisms (SNPs) within miRNA genes or miRNA target sites could result in their functional changes. Therefore, we hypothesized that SNPs in miRNAs and/or their target sites were associated with schizophrenia. 3 SNPs in *hsa*-*pri-miR-219/132/107* and 6 SNPs in 3′UTRs of *GRIN2A/2B/3A* and *CAMK2G* were selected and genotyped in a case-control study of 1041 schizophrenia cases and 953 healthy controls in Chinese Han population. In the present study, *GRIN2B* rs890 showed significant associations with schizophrenia. Further functional analyses showed that the rs890 variant C allele led to significantly lower luciferase activity, compared with the A allele. MDR analysis showed that a 4-locus model including rs107822, rs2306327, rs890 and rs12342026 was the best model. These findings suggest that *GRIN2B* may be associated with schizophrenia and interaction effects of the polymorphisms in *hsa-miR-219*, *CAKM2G*, *GRIN2B* and *GRIN3A* may confer susceptibility to schizophrenia in the Chinese Han population.

Schizophrenia (SCZ) is a complex mental disorder with a population prevalence of about 1%, which is characterized by dysregulation of multiple signaling pathways, including dopaminergic signaling, glutamatergic signaling, neuroplasticity signaling etc^1^. Recent evidence suggests that microRNAs (miRNAs) have potential role in the pathophysiology of SCZ^2^,^3^.

MiRNAs are small noncoding single-stranded RNA molecules of 21–23 nt in length, which function as critical regulators of genes expression by binding to the 3′ untranslated regions (3′UTR) of the target mRNAs and triggering the cleavage or translational repression of these targets. Most miRNAs were derived from a two-step sequential processing: the generation of pre-miRNA (60–70 nucleotide long) from pri-miRNA (500–3000 bases long) by the Drosha/DGCR8 complex in the nucleus, and the generation of mature miRNAs(21–25 bases long) from pre-miRNAs by the Dicer/TRBP complex in the cytoplasm^4^. Several post-mortem studies have suggested that plenty of miRNAs were dysregulated in SCZ and therefore modulated the expression of many genes, several of which have been described to be related to SCZ^5^. Pathway analyses have highlighted nervous system development and processes involved in synaptic transmission as major effect points of altered miRNA expression. The ability of miRNAs controlling their target mRNAs or regulating the protein levels of some signaling pathways indicated they had important implications for SCZ^6^.

Disturbances in glutamatergic neurotransmission, particularly caused by dysfunction or dysregulation of N-methyl-D-aspartate-type glutamate receptor (NMDAR) is regarded as the final common pathway on the road to SCZ^7^. Several findings suggested that miR-219, miR-132, and miR-107 had important roles in regulating NMDAR-induced calcium signaling pathway^6^ (Fig. 1).

MiR-219, miR-132, and miR-107 are abundant in human brain. Kocerha *et al.*^8^ found that miR-219 mediated the behavioral effects of MK-801 treatment in mice and targeted *CAMK2G*, a member of the calcium/calmodulin-dependent protein kinase family involved in NMDAR signaling. Other study showed that miR-219 could attenuate NMDA-induced neuronal depolarization^9^ and was upregulated in dorsolateral prefrontal corte (DLPFC) from schizophrenic patients^10^. These results indicated that miR-219 might inhibit NMDAR signaling at the levels of both the receptor and second messenger signaling.

Findings indicated that miR-132 could enhance activity-dependent synaptic plasticity through a positive feedback loop with the NMDAR: it was both induced by, and potentiated, NMDAR signaling^9^,^11^. Miller *et al.* observed that miR-132 was significantly down-regulated in schizophrenic subjects. Analysis of miR-132 target gene expression in schizophrenia gene-expression microarrays identified 26 genes up-regulated in schizophrenia subjects^12^. Consistent with NMDA-mediated hypofunction observed in schizophrenic subjects, administration of an NMDA antagonist to adult mice resulted in the down-regulation of miR-132 expression and up-regulation of several miR-132 targets in the adult PFC^12^. Study using miRanda showed that the predicted *miR-132* gene targets could interact with SCZ candidate genes (*GRIN1*, *GRIN2A*, *GRIN2B*, *BDNF* and *DRD1*)^13^.

MiR-107 was upregulated in DLFC from schizophrenic patients^14^ and was identified to have a strong regulation effect on 3′UTR elements from NMDAR subunit 3A (*GRIN3A*) and was highly enriched in pathways involved in neural connectivity and synaptic plasticity, such as axon guidance, longterm potentiation, Wingless/int (Wnt), epidermal growth factor receptor family (ErbB) and Mitogenactivated protein (MAP) kinase signaling. These processes were repeatedly implicated in the pathophysiology of schizophrenia^14^.

Although the aforementioned studies suggested a link between miRNA dysregulation and NMDAR signaling in SCZ, the specific mechanism of interaction between them is still unknown. Polymorphisms that may potentially affect miRNA mediated regulation of the cell can be present not only in the 3′UTR of a miRNA target gene, but also in the genes involved in miRNA biogenesis and in pri-, pre- and mature-miRNA sequences. A polymorphism in processed miRNAs may affect expression of several genes and have serious consequences, whereas a polymorphism in miRNA target site, in the 3′UTR of the target mRNA,may be more target and/or pathway specific^15^. In this study, we hypothesized that single nucleotide polymorphisms (SNPs) in *hsa-miR-219, hsa-miR-132* and *hsa- miR-107* genes and 3′UTR of NMDAR signaling pathway genes (*GRIN1, GRIN2A, GRIN2B, GRIN3A and CAMK2G*) may alter the expression of these miRNAs or their target genes and/or maturation of miRNAs, and therefore individually and/or jointly contribute to SCZ. To test this hypothesis, we performed an association analysis for 3SNPs in *hsa*-*pri-miR-219/132/107* and 6 SNPs in 3′UTR of NMDAR signaling pathway genes in a case-control study of 1041 SCZ cases and 953 healthy controls in Chinese Han population.

## Materials and Methods

### Participants

In the case–control study, 1041 patients with SCZ (531 males and 510 females; mean age: 33.65 ± 9.14years) were recruited from Shanghai Mental Health Center according to the criteria of the Diagnostic and Statistical Manual of Mental Disorders, Fourth Edition (DSM-IV). 953 healthy controls (512 males and 441females; mean age: 34.47 ± 10.49 years) were recruited from urban community, as well as staff members at Shanghai Mental Health Center. All of the participants were Chinese Han descendants and do not have the history of mental and physical illness. This study was approved by the ethical committee of Shanghai Mental Health Center (Approval number: 2008–25) and signed informed consent forms were obtained from all the subjects. The experiments were performed according to the regulations and guidelines established by this committee.

### SNP selection and genotyping

**Figure 2:**
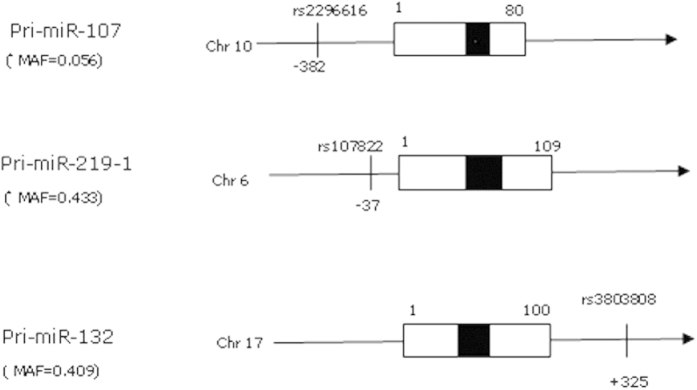
The locations of 3 SNPs in the pri-miRNA transcripts. The locations of SNPs in pri-miRNAs are depicted on black line at the upstream (negative base count, ‘–’) or downstream (positive base count, ‘+’) of the pre-miRNA. The figure is not drawn to scale.

We selected 3 SNPs (rs107822, rs3803808, rs2296616) within +/−500 bp of *hsa-pre-miR-219, hsa-pre-miR-132* and *hsa-pre-miR-107* transcripts and 6 SNPs(rs1420040, rs1805502, rs890, rs16920487, rs12342026, rs2306327) in 3′UTR of NMDAR signaling pathway genes (*GRIN1, GRIN2A, GRIN2B, GRIN3A and CAMK2G*) from hapmap database (release #24) in Chinese Han population (minor allele frequency, MAF ≥ 0.05). According to the PolymiRTS Database 3.0 (a database of naturally occurring DNA variations in miRNA seed regions and miRNA target sites, http://compbio.uthsc.edu/miRSNP/home.php) and the patrocles (a database of polymorphic miRNA-target interation, http://www.patrocles.org/). rs1420040, rs890, rs16920487, rs12342026, rs2306327 were in the putative miRNA target sites. There was no SNPs in *GRIN1* which meet our screening requirements. The locations of SNPs in the pri-miRNA were showed in Fig. 2. The information of SNPs in 3′UTR were showed in Table 1.

Genomic DNA was extracted from peripheral blood according to standard laboratory procedures (Blood genomic DNA extraction kit, TIANGEN, Beijing). All of the 9 SNPs were genotyped with the TaqMan genotyping method according to the manufacturer’s protocol, where allelic discrimination and analysis were performed using an ABI Prism 7900 Sequence Detection System (Applied Biosystems, Foster City, CA). Primers and probes were purchased from Applied Biosystems.

For quality control, all genotypes were called blind to the case or control status in the genotyping process. Of the samples collected, 5% were repeated for the genotyping assay, and the results were more than 99% concordant.

### Luciferase Constructs for the 3′UTR of the Human *GRIN2B*

Luciferase plasmids were constructed as follows. Primers were designed to amplify the 3′ UTR of GRIN2B containing A allele at rs890 in humans with tags introducing the XbaI and HpaI restriction enzyme site by using forward primer 5′-gctctagaGTGAGGGAACAGAGAGGTTAAGGTG-3′ and reverse primer 5′-gccaattgGTTTTGGGGGGAAGAAGCCTG-3′. After amplification, PCR product was digested by XbaI and HpaI and was inserted into the pGL3-promoter firefly luciferase reporter vector (Promega) between its XbaI and HapI sites (pGL3-promoter-A). Mutant reporter plasmid containing C allele at rs890 (pGL3-promoter-C) was generated using the pGL3-promoter-A as a template and the QuickChange site-directed mutagenesis kit (Stratagene) according to the manufacturer’s instructions. The primers for mutegenesis were 5′-GGTAGCTTTTCCCAAACGGATCTTTTCATTTAGG-3′(F) and 5′- CCTAAATGAAAAGATCCGTTTGGGAAAAGCTACC-3′ (R). Both constructs were sequence verified prior to use.

### Luciferase assay

Human embryonic kidney (HEK) 293 cells were cultured in Dulbecco’s modified Eagle’s medium with 10% fetal bovine serum, 100 U/ml penicillin, 100 U/ml streptomycin at 37 °C in the presence of 5% CO2. The cells were transiently cotransfected with pRL-SV40 Renilla reporter vector (Promega) as an internal control and pGL3-promoter-A or pGL3-promoter-C using FuGENE HD Transfection Reagent (Roche) according to the manufacturer’s protocol at 80–90% confluence in a 24-well culture dish. The control plasmid contained the pGL3 promoter, rather than the *GRIN2B* 3′UTR. This control was cotransfected with the pRL-SV40 Renilla reporter vector as a positive control for luciferase expression. The cells were harvested 24 hours after transfection and the activities of both the firefly luciferase and Renilla luciferase were measured using the Dual-Luciferase Reporter Assay System (Promega) on Berthold LB940 according to the manufacturer’s protocols. The relative firefly luciferase activities were normalized against the Renilla luciferase activities.

### Statistical analysis of polymorphisms

Test of Hardy–Weinberg (H-W) equilibrium in control samples and the distributions of the allele and genotype frequencies were performed by SHEsis (http://analysis.bio-x.cn/SHEsisMain.htm)^16^.

Multifactor Dimensionality reduction (MDR) software (version 2.0) was employed to detect the interactive effect between the genes and select the best multi-locus interactive model^17^,^18^,^19^. First, we selected the top four polymorphisms for interaction analysis using the Tuned ReliefF (TuRF) filter algorithm of Moore and White^20^. Second, we constructed all possible combinations of one, two, three, and four polymorphisms using MDR constructive induction algorithm. Third, we used a naı¨ve Bayes classifier in the context of 10-fold cross-validation to estimate the testing accuracy of each best one-,two-, three-, and four- factor model. A single best model was selected that maximized the testing accuracy. We also report the cross-validation (CV) consistency that measures the number of times of 10 divisions of the data that the same best model was found. Statistical significance was evaluated using a 1000-fold permutation test to compare observed testing accuracies with those expected under the null hypothesis of no association. Permutation testing corrects for multiple testing by repeating the entire analysis on 1000 datasets that are consistent with the null hypothesis. Models were considered significant at the 0.05 levels.

The effect of polymorphisms of rs890 on expression levels was tested by one-way analysis of variance (ANOVA) using GraphPad Prism 5. Values were presented as mean ± SEM.

For all associations, a P value of  <0.05 was considered as statistically significant. The Bonferroni correction was applied by multiplying P values by the number of SNPs analyzed (n = 7)^21^.

## Results

To examine the association between schizophrenia and 9 SNPs, we carried out a case-control study recruiting 1041 schizophrenia patients and 953healthy individuals. Because the observed genotype frequencies for 2 SNPs (rs1805502 and rs16920487) in the controls were not in Hardy–Weinberg equilibrium (P =  0.006 for rs1805502 and P = 0.031 for rs16920487). These 2 SNPs were not included in the following analysis.

### Single SNP analysis

The genotypes and alleles frequencies of the 7 SNPs were present in Table 2. Significant differences were found in allele and genotype frequencies between patients and controls at 2 SNPs, rs107822 in *hsa-miR-219* (p = 0.007 for allele;p = 0.027 for genotype) and rs890 in *GRIN2B* (p = 0.005 for allele;p = 0.001 for genotype). After Bonferroni correction, significant association between the allele and genotype only remained in rs890 (P = 0.035 and 0.007, respectively).The fact that the frequencies of allele C (OR = 0.81, 95%CI = 0.70–0.94) and genotype CC (OR = 0.46, 95%CI = 0.30–0.70) at rs890 were lower in patient group than in control group suggested that individuals carrying alleles C could decrease the risk of developing SCZ.

### Gene-gene interaction analysis

The four best polymorphisms selected by TuRF for interaction analysis included *hsa-miR-219* rs107822, *GRIN3A* rs12342026, *GRIN2B* rs890 and *CAMK2G* rs2306327. Table 3 summarized the results of the MDR analysis that evaluated all possible combinations of these four polymorphisms. Of the four models, the 4- locus model that including rs107822, rs12342026, rs890 and rs2306327 had a a maximum CV consistency (10/10) ((1000-fold permutation p < 0.0001) and was considered as the best model. In the χ^2^ test, the OR of high-risk combination of 4 loci showed an increase of the risk of schizophrenia by 2.35 times (OR = 2.35, 95% CI = 1.94–2.85) when compared with participants who had a ‘‘low risk’’ SNP combination.

### The effect of SNP rs890 on GRIN2B

**Figure 3:**
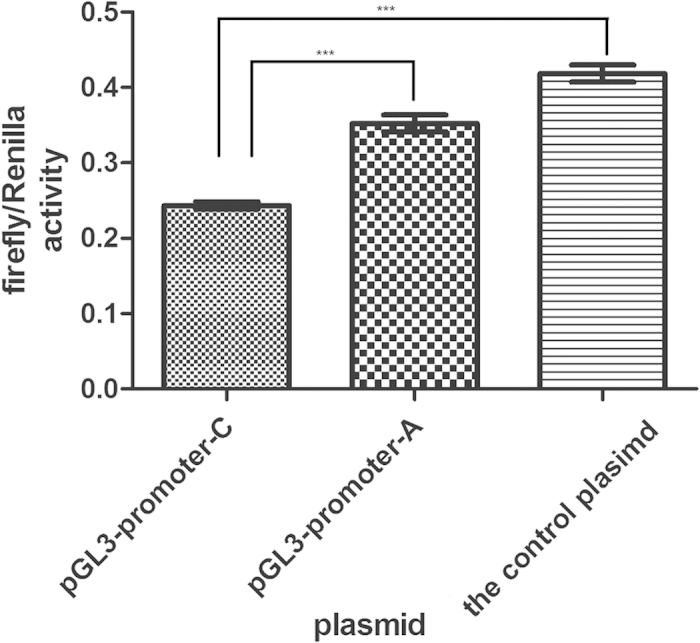
The luciferase activity of the three constructs transfected in HEK293 were graphed after normalization against the plasmid pRL-SV40 expressing the Renilla luciferase gene. Data of the control plasimid (contained the pGL3 promoter, rather than the *GRIN2B* 3′UTR.) is presented as mean with SEM of n = 6 samples. Data of both 3′UTR constructs (pGL3-promoter-A and pGL3-promoter-C) are presented as means with SEM of n = 5 sample espectively. Comparisons were determined using ANOVA with post-hoc comparisons ***p < 0.001, One-way ANOVA test. Error bars are standard error.The luciferase activities of 3′-UTR constructs were significantly (p < 0.001) reduced compared with the pGL3 -Promoter vector 24 h after transfection, A mutant-type 3′-UTR construct (pGL3-promoter-C) showed lower (p < 0.001) activity than the wild-type 3′-UTR construct (pGL3-promoter-A). The relative firefly luciferase activities were normalized against the Renilla luciferase activities.

To analyze the effect of SNP rs890 on *GRIN2B*, the 3′ UTR of human *GRIN2B* was cloned and inserted into the pGL3-promoter firefly luciferase vector. Two constructs carrying A or C at rs890 (pGL3-promoter-A, pGL3-promoter-C) were generated through site directed mutation and were transfected into HEK293 cells. Table 4 showed the relative firefly luciferase activities which were normalized against the Renilla luciferase activities. As shown in Fig. 3, the luciferase activity of pGL3-promoter-C (mean:0.244, SEM:0.005) was about 11% lower than that of pGL3-promoter-A (mean:0.353, SEM:0.011)(P < 0.001), while the luciferase activities of both constructs were significantly lower than the pGL3-promoter vector (the control plasmid, mean:0.419, SEM:0.011) (P < 0.001).

## Discussion

NMDA receptors are heteroligomers with multiple subunits surrounding a central pore that serves as an intrinsic ion channel. Each subunit has different functions. Although the mechanistic basis for NMDAR hypofunction in SCZ remains unknown, it could be caused by a change in NMDAR subunit composition. In addition, miRNAs could also influence the normal expression of NMDAR subunits or transmission of NMDAR signaling . In this case–control study, we selected 6 SNPs in the 3′UTR of NMDAR signaling pathway genes and 3 SNPs in *hsa-miR-219*, *hsa-miR-107 a*nd *hsa-miR-132*. Association analysis suggested that rs107822 in *hsa-miR-219* were nominally associated with SCZ and rs890 in *GRIN2B* were significantly associated with SCZ.

Pri-miRNA processing is a critical step in miRNA biogenesis because it defines the miRNA sequences embedded in long pri-miRNAs by generating one end of the molecule. Drosha -DGCR8 complex plays an essential roles in pri-miRNA processing. An ‘‘ssRNA-dsRNA Junction Anchoring’’ model for the processing of pri-miRNA has been proposed. The cleavage site is determined mainly by the distance (_11 bp) from the stem-ssRNA junction. DGCR8 interacts with pri-miRNAs directly and the flanking ssRNA segments are vital for this binding to occur^22^. According to this model, the flanking ssRNA segments are critical for processing and if a mutation causes a structural change in the crucial regions of miRNA, the mutation could affect the processing and the expression of the miRNA. rs107822(T > C) is located 37 nt downstream of the pre-miR-219 sequence and it may have some effects on miR-219 processing. However, the processing and maturation of miRNA *in vivo* is complicated. Some mutations that were close or even in the miRNA precursor were predicted to cause major conformational changes of the miRNAs, but none of these changes affected the processing or expression of the mature miRNAs *in vivo*^23^. In contrast, in allele variants with no predicted secondary structural changes, such as Gt19A mutation in the let-7e identified herein^24^ and reported Ct7T substitution in miR-16-1^25^, the expression of their mature miRNAs were remarkably reduced *in vivo*. It is also possible that the environments surrounding miRNA and unknown factors that interact with the miRNA might affect its folding *in vivo* or protect its structural conformation against changes caused by some mutations.

Regardless of the genetic mechanism involved in associations between *miR-219* and SCZ, *miR-219* is known to be expressed and has important functions in the brain. It is located at 6p21,which is a putative susceptibility locus for schizophrenia. It plays a role in circadian rhythms^9^ and appears to be an integral component of the NMDAR signaling cascade^8^.

Other than serving as a miRNA binding site, the 3′UTR harbors the hexanucleotide AAUAAA signal for cleavage, polyadenylation and cis-acting elements. Polymorphisms in the 3′UTR could influence gene expression, polyadenylation site selection and mRNA stability^26^.

Previous association evidence showed *GRIN2B* was significantly associated with SCZ and significant gene region association was found for the 3′ region^27^,^28^,^29^,^30^. For example, a mutation analysis of *GRIN2B* in Japanese population found that only rs7301328(366C/G) polymorphism in the 3′ region of the last exon was statistically significant assosiated with schizophrenia. Three polymorphisms (5806A/C, rs1805502, 4165C/T) in 3′UTR were deviated from Hardy–Weinberg equilibrium^30^. In our study, rs1805502 was also deviated from Hardy–Weinberg equilibrium. The possible explannation included mate choice, mutation, selection, genetic drift, gene flow and meiotic drive etc. This SNP was not included in the following analysis. An Italian case-control study found a marginally significant excess of homozygosity for the rs890^29^. An Danish study found 9 SNPs (rs890, rs1806191, rs10772692, rs2270359, rs1806194, rs1806195, rs1805482, rs1805199 and rs1805554) out of 30 in *GRIN2B* were significantly associated with schizophrenia population. Moreover, all of the 9 SNPs except one (rs180554) were located at the 3′region of the gene. A gene-wise association test showed significant gene–region association was only found for the 3′region of the gene. The level of LD between the genotyped SNPs at the 3′region of the gene is low, indicating the presence of multiple risk variants^28^. The result of significant association with schizophrenia for rs890 in an Danish sample study^28^ (allele C: OR = 0.869, P = 0.0127) was consistent with our finding (allele C: OR = 0.81, P = 0.005 ). Our association study suggested that rs890 might confer susceptibility to schizophrenia. Interestingly, according to PolymiRTS Database 3.0 (http://compbio.uthsc.edu/miRSNP/home.php), a database of naturally occurring DNA variations in microRNA (miRNA) seed regions and miRNA target sites, rs890 is in a putative targeted site. The A allele at rs890 could disrupt a conserved miRNA site (miR-4328) and cause loss of normal repression control. The C allele could create a new miRNA site (miR-1468-5p)and cause abnormal gene repression. Rs890 is most likely to have functional impacts. Further functional analyses showed that the rs890 variant C allele led to significantly lower luciferase activity, compared with the A allele.

*GRIN3A* encodes NMDAR subunit 3A (NR3A), which plays an important role in modulating the NMDAR activity^31^. In a post-mortem study of schizophrenic patients, NR3A expression level was found to be selectively elevated in the gyri of the DLPFC, indicating its involvement in the pathophysiology of schizophrenia^32^. However, in our study *GRIN3A* (rs12342026 in 3′UTR) was not significantly associated with SCZ , suggesting an absence of an association between genetic variants in putative regulatory subunit of the *GRIN3A* gene (for example rs1337676 at the 5′UTR and rs1983812 at the 3′UTR) and SCZ^33^,^34^.

Because of the small effect size of single SNP on complex disease, We hypothesized that gene–gene interaction probably contributed to the pathogenesis of SCZ. Using MDR, we found a significant interaction of four locus model including rs107822 (*hsa-miR-219*), rs2306327 (*CAMK2G)*, rs890 (*GRIN2B*) and rs12342026 (*GRIN3A*). In this model, only rs890 was significant associated with SCZ, supporting the following view: schizophrenia is a complex and multigenetic disease. The occurrence and prognosis for schizophren and other complex diseases is most likely controlled by a combination of multiple genetic factors and exposures, rather than a single polymorphism.

In summary, our study showed that polymorphism in *GRIN2B* may be associated with susceptibility to SCZ and provides the possibility that the interaction effects of the polymorphisms in *has-miR-219, CAMK2G, GRIN2B,* and *GRIN3A* are associated with SCZ in the Chinese population. However our findings need to be replicated on larger or different population samples.

## Additional Information

**How to cite this article**: Zhang, Y. *et al.* Polymorphisms in MicroRNA Genes And Genes Involving in NMDAR Signaling and Schizophrenia: A Case-Control Study in Chinese Han Population. *Sci. Rep.* **5**, 12984; doi: 10.1038/srep12984 (2015).

## Figures and Tables

**Figure 1:**
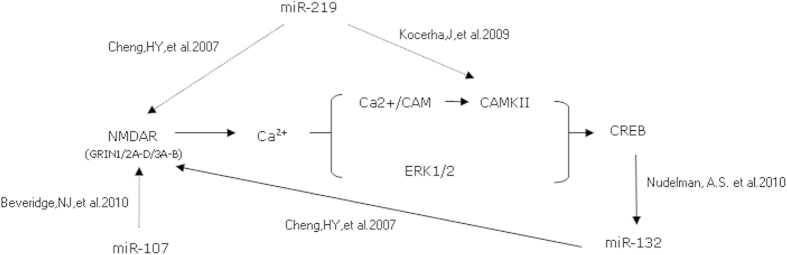
Possible roles of miRNAs in NMDA signaling pathway associated with SCZ.

**Table 1:** SNPs in 3′UTR of NMDARs and CAMK2G genes (GRch38).

Gene	3′UTR length	SNPs in 3′UTR	Chr. Location	MAF
GRIN3A	3821 bp	rs16920487 (A > T)	9:101571102	0.068
		rs12342026 (G > A)	9:101571582	0.284
GRIN2A	9739 bp	rs1420040 (A > G)	16:9756540	0.411
GRIN2B	1307 bp	rs1805502 (A > G)	12:13561247	0.212
		rs890 (A > C)	12:13562374	0.23
CAMK2G	2017 bp	rs2306327 (A > T)	10:73814020	0.102

**Table 2:** The distribution of alleles and genotypes for 7 SNPs in SCZ and control subjects.

SNP	Case N(freq)	Control N(freq)	OR (95%CI)	P-value	P-value*
rs2296616(A > G) (*pri-miR-107*)	1034	935			
G	141(0.068)	155(0.083)	0.81(0.64–1.03)	0.080	0.560
A	1927(0.932)	1715(0.917)	1.24(0.97–1.57		
AA	898(0.868)	784(0.838	1.00	0.150	1.050
AG	131(0.127)	147(0.157)	0.78(0.60–1.00)		
GG	5(0.005)	4(0.004)	0.78(0.29–4.08)		
rs107822(T > C) (*pri-miR-219*)	1035	921			
C	739(0.357)	735(0.399)	**0.84(0.73**–**0.95)**	**0.007**	0.049
T	1331(0.643)	1107(0.601)	**1.19(1.05**–**1.37)**		
TT	432(0.417)	335(0.364)	1	**0.027**	0.189
CT	467(0.451)	437(0.475)	0.83(0.68–1.01)		
CC	136(0.131)	149(0.162)	**0.71(0.54**–**0.93)**		
rs3803808(G > A) (*pri-miR-132*)	1021	938			
A	1074(0.526)	955(0.509)	1.07(0.94–1.21)	0.290	2.030
G	968(0.474)	921(0.491)	0.93(0,83–1.06)		
GG	236(0.231)	239(0.255)	1.00	0.474	3.318
AG	496(0.486)	443(0.472)	0.99(0.80–1.23)		
AA	289(0.283)	256(0.273)	0.87(0.68–1.12)		
rs1420040(A > G) (*GRIN2A*)	1036	920			
G	862(0.416)	767(0.417)	1.00(0.88–1.14)	0.958	6.706
A	1210(0.584)	1073(0.417)	1.00(0.88–1.14)		
AA	354(0.342)	314(0.341)	1.00	1.000	7.000
AG	502(0.485)	445(0.484)	1.00(0.82–1.22)		
GG	180(0.174)	161(0.175)	0.99(0.76–1.29)		
rs890(A > C) (*GRIN2B*)	1038	938			
C	457(0.220)	484(0.258)	**0.81(0.70**–**0.94)**	**0.005**	**0.035**
A	1619(0.780)	1392(0.742)	**1.23(1.06**–**1.43)**		
AA	617(0.594)	520(0.554)	1.00	**0.001**	**0.007**
AC	385(0.371)	352(0.375)	0.92(0.77–1.11)		
CC	36(0.035)	66(0.007)	**0.46(0.30**–**0.70)**		
rs12342026(G > A) (*GRIN3A*)	1029	928			
A	502(0.244)	491(0.265)	0.90(0.78–1.04)	0.139	0.973
G	1556(0.756)	1365(0.735)	1.11(0.96–1.28)		
GG	595(0.578)	494(0.532)	1.00	0.071	0.497
AG	366(0.356)	377(0.406)	0.81(0.67–0.97)		
AA	68(0.066)	57(0.061)	0.99(0.68–0.99)		
rs2306327(A > T) (*CAMK2G*)	1039	935			
T	225(0.108)	203(0.109)	1.00(0.81–1.22)		
A	1853(0.892)	1667(0.891)	1.00(0.82–1.23)	0.978	6.846
AA	822(0.791)	743(0.795)	1.00		
AT	209(0.201)	181(0.194)	0.04(0.84–1.30)	0.608	4.256
TT	8(0.008)	11(.0.12)	0.66(0.26–1.64)		

Bold value indicates significant result (P < 0.05).

P* is corrected by Bonferroni correction.

**Table 3:** MDR interaction models for schizophrenia.

No. of loci in model	SNPs included in the best combination in each model	Training accuracy	Testing accuracy	CV consistency	OR(95%CI)^a^	P value^b^
1	rs107822	0.5332	0.5086	6/10	1.31(1.09–1.57)	0.0036
2	rs107822, rs890	0.5530	0.5460	9/10	1.78(1.38–3.04)	<0.0001
3	rs107822, rs2306327, rs12342026	0.5712	0.5308	8/10	1.83(1.52–2.20)	<0.0001
4	rs107822,rs2306327,rs890,rs12342026	0.5922	0.5506	10/10	2.35(1.94–2.85)	<0.0001

^a^Odds ratio represents the odds of schizophrenia in the ‘‘high risk’’ group compared to the ‘‘low risk’’ group identified by MDR.

^b^P value is corrected by a 1000-fold permutation test.

**Table 4:** The relative firefly luciferase activities were normalized against the Renilla luciferase activities.

sample	firefly/Renilla activity		
	pGL3-promoter-C	pGL3-promoter-A	pGL3-promoter
1	na	na	0.466 (1223800/2626100)
2	0.241(199510/828420)	0.325 (151250/465040)	0.388 (1400200/3608500)
3	0.244(255660/1047900)	0.326 152230/466620)	0.414(578670/1399100)
4	0.255 387070/1517500)	0.365 139790/382850)	0.414 (677260/1635100)
5	0.251 379830/1512200)	0.380(207090/545180)	0.399(894590/2242900)
6	0.227(435650/1915700)	0.366(84295/230320)	0.430 (1902800/4420000)
